# ALK Expression Without ALK Rearrangement in EWSR1::ATF1-Fused Inflammatory and Nested Testicular Sex Cord Tumor

**DOI:** 10.3390/diagnostics16172847

**Published:** 2026-09-04

**Authors:** Min Chong Kim, Hee Jung Kwon, Su Hong Kim

**Affiliations:** 1 Department of Pathology, Yeungnam University Medical Center, Daegu 42415, Republic of Korea; wowlow2@gmail.com; 2 Department of Pathology, College of Medicine, Yeungnam University, Daegu 42415, Republic of Korea; hjs336@ynu.ac.kr; 3 Department of Radiology, College of Medicine, Yeungnam University, Daegu 42415, Republic of Korea

**Keywords:** testicular tumor, sex cord–stromal tumor, *EWSR1::ATF1* fusion, CD30, ALK, inflammatory and nested testicular sex cord tumor

## Abstract

Inflammatory and nested testicular sex cord tumor (IN-TSCT) is a recently recognized, rare testicular sex cord–stromal neoplasm characterized by a recurrent *EWSR1::ATF1* fusion and potentially aggressive clinical behavior. Because of its nested epithelioid morphology accompanied by prominent inflammatory infiltrates, IN-TSCT may be mistaken for seminoma, while frequent diffuse CD30 expression further complicates the differential diagnosis with lymphoma. We report a case of IN-TSCT in a 54-year-old man presenting with intermittent left testicular pain. Scrotal ultrasonography demonstrated a well-defined, heterogeneously hypoechoic intratesticular mass with minimally increased internal vascularity. Radical orchiectomy revealed a 1.5 × 1.4 cm epithelioid neoplasm arranged in nests and cords with a prominent inflammatory infiltrate. The tumor cells expressed the sex cord–stromal markers SF-1 and calretinin and showed diffuse CD30 expression. RNA-based next-generation sequencing identified an *EWSR1::ATF1* fusion, establishing the diagnosis of IN-TSCT. Unexpectedly, the tumor also demonstrated strong ALK immunoreactivity with both the ALK1 and D5F3 antibody clones, despite the absence of an ALK fusion by RNA sequencing or an ALK rearrangement by fluorescence in situ hybridization. The combined expression of CD30 and ALK may mimic anaplastic large cell lymphoma and represents an important diagnostic pitfall. The patient remained free of recurrence or metastasis 18 months after orchiectomy. This case expands the recognized immunophenotypic spectrum of IN-TSCT and demonstrates that strong ALK immunoreactivity does not necessarily indicate an underlying ALK fusion or rearrangement. Given the potentially aggressive clinical behavior reported in IN-TSCT, long-term oncologic surveillance is warranted.

**Figure 1:**
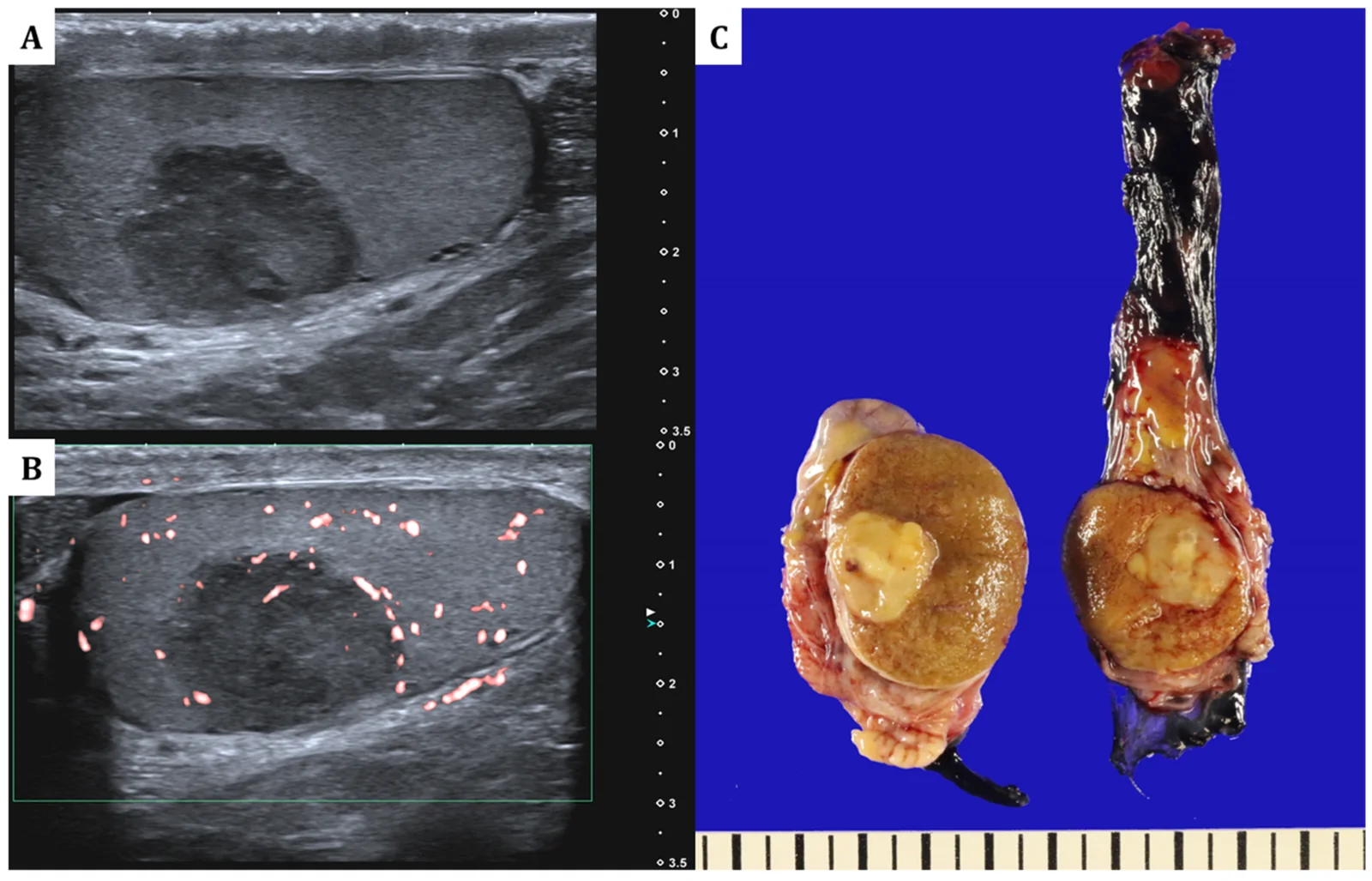
A 54-year-old man presented with a 3-month history of dull, intermittent pain in the left testis, rated 2/10 on the numeric rating scale. Serum tumor markers were within normal limits, including alpha-fetoprotein (AFP), 2.9 ng/mL (reference range, 0.56–7.56 ng/mL), and β-human chorionic gonadotropin (β-hCG), <0.2 mIU/mL. Gray-scale ultrasonography demonstrated a well-defined, heterogeneously hypoechoic intratesticular mass in the left testis (**A**). Superb microvascular imaging showed minimally increased internal vascularity (**B**). The patient subsequently underwent left radical inguinal orchiectomy. Gross examination revealed a well-circumscribed, tan-white intratesticular mass measuring 1.5 × 1.4 cm, without extratesticular extension (**C**; thick ruler markings indicate 1 cm intervals).

**Figure 2:**
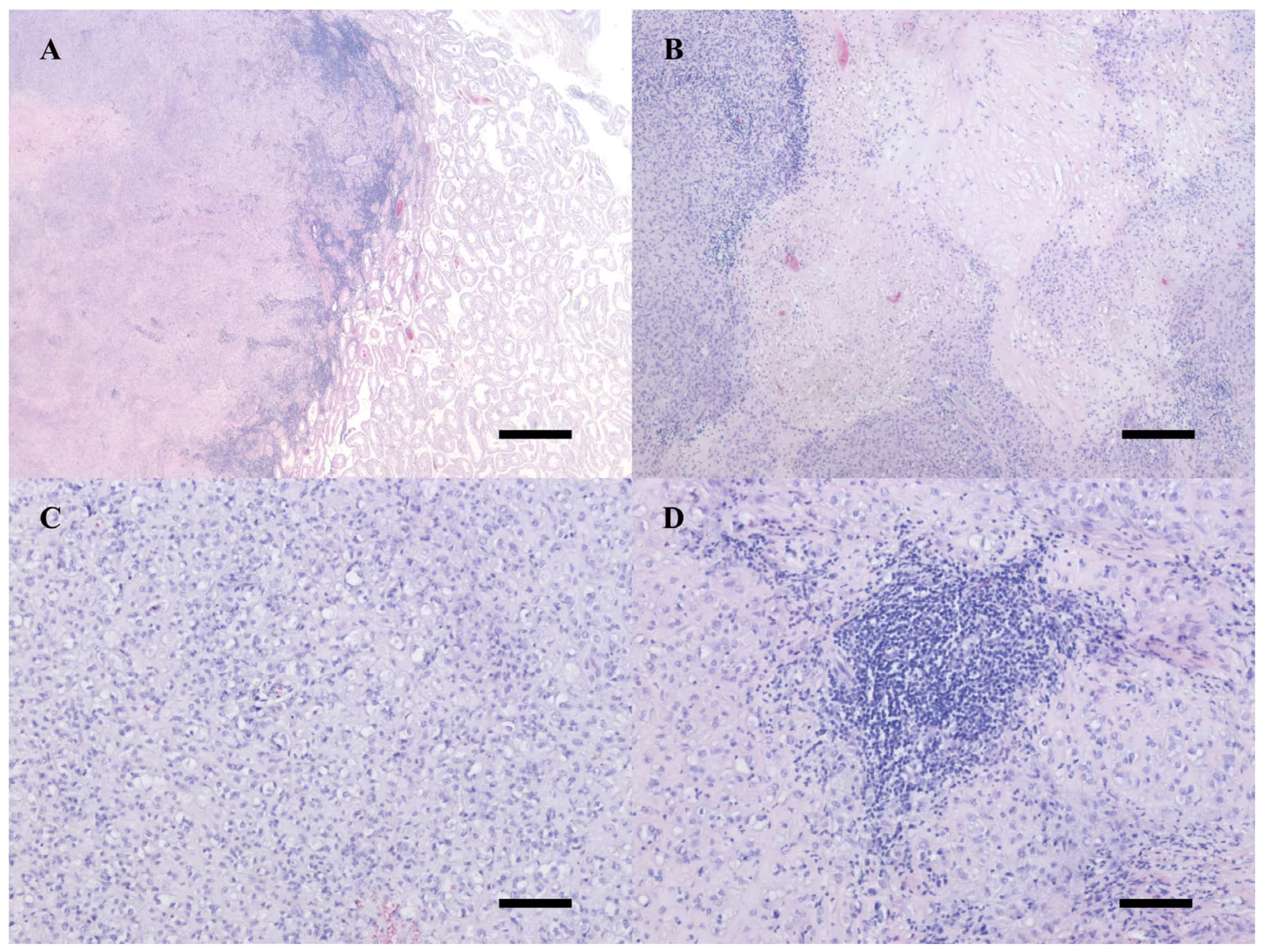
Although sharply circumscribed, the tumor lacked a fibrous capsule and showed prominent peripheral lymphoid cuffing (**A**; hematoxylin and eosin [H&E] stain; original magnification, ×10; scale bar = 1000 μm). At intermediate magnification, the tumor was composed predominantly of sheets and focal nests of epithelioid cells. Small foci of hyalinized stroma and focal necrosis were present mainly in the central portion of the tumor (**B**; H&E stain; ×40; scale bar = 250 μm). The tumor cells had abundant eosinophilic cytoplasm with focal cytoplasmic vacuolization (**C**; H&E stain; ×100; scale bar = 100 μm). The tumor cells had irregular nuclei with vesicular chromatin and conspicuous nucleoli. Patchy intratumoral aggregates of inflammatory cells, composed predominantly of lymphocytes with scattered plasma cells, were present throughout the tumor (**D**; H&E stain; ×100; scale bar = 100 μm). The highest mitotic count was 4 mitoses per 2 mm^2^. The combination of epithelioid cytology, nested or sheet-like architecture, peripheral lymphoid cuffing, and intratumoral inflammatory infiltrates raised the possibility of IN-TSCT, although seminoma and lymphoma remained important morphologic mimics.

**Figure 3:**
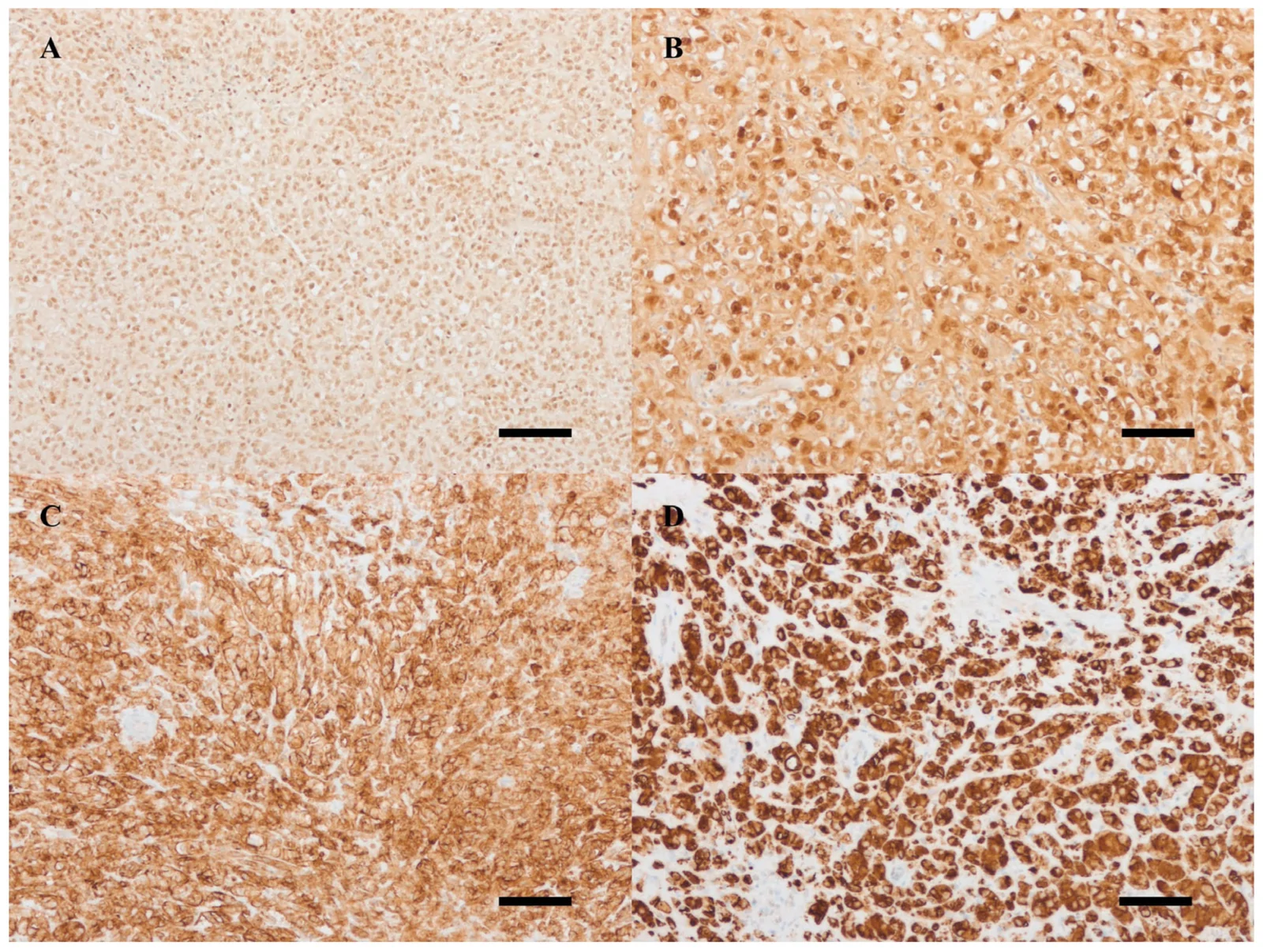
Immunohistochemical staining demonstrated diffuse nuclear expression of steroidogenic factor 1 (SF-1) (**A**; original magnification, ×100; scale bar = 100 μm) and diffuse cytoplasmic expression of calretinin (**B**; ×100; scale bar = 100 μm), supporting sex cord–stromal differentiation. The tumor also showed diffuse CD30 expression (**C**; ×100; scale bar = 100 μm), together with EMA positivity and focal reactivity for cytokeratins AE1/AE3 and CAM5.2. Of particular note, ALK immunoreactivity was observed with both the ALK1 and D5F3 antibody clones, with stronger staining using D5F3 (**D**; ×100; scale bar = 100 μm). The tumor lacked expression of the lymphoid markers LCA, CD3, CD20, and granzyme B, as well as the germ cell markers SALL4, β-hCG, AFP, PLAP, and CD117. Targeted DNA/RNA-based next-generation sequencing identified an *EWSR1::ATF1* fusion, supported by 5168 reads, with a variant allele frequency of 10.5%, consistent with the recurrent molecular alteration reported in IN-TSCT. No *ALK* fusion was identified by RNA-based next-generation sequencing, and the absence of *ALK* rearrangement was further confirmed by *ALK* break-apart fluorescence in situ hybridization. Postoperative 18F-FDG PET/CT showed no evidence of nodal or distant metastatic disease. IN-TSCT is a recently recognized, rare testicular sex cord–stromal neoplasm characterized by a recurrent *EWSR1::ATF1* fusion and potentially aggressive clinical behavior [1]. The principal educational significance of this case lies not merely in the rarity of IN-TSCT, but in the potential diagnostic pitfall created by strong ALK immunoreactivity in the absence of an underlying ALK rearrangement. Because of its rarity and the limited number of reported cases, IN-TSCT remains an underrecognized entity and may be misdiagnosed as seminoma [1,2]. Although its ultrasonographic appearance is nonspecific, IN-TSCT should be considered in testicular tumors showing nested epithelioid morphology, prominent inflammatory infiltrates, and a distinctive immunophenotype characterized by coexpression of sex cord–stromal markers, EMA, and CD30 in the absence of germ cell marker expression. This combination of findings should prompt molecular evaluation for an *EWSR1::ATF1* fusion [2]. In a testicular tumor showing concurrent CD30 and ALK expression, careful correlation with morphology and a focused immunohistochemical panel including lymphoid, germ cell, and sex cord–stromal markers is essential. When the morphologic and immunophenotypic findings are discordant, molecular testing for characteristic gene fusions, particularly EWSR1::ATF1, should be considered. In the present case, strong ALK immunoreactivity together with diffuse CD30 expression in the epithelioid tumor cells initially led to diagnostic confusion with other neoplasms, particularly anaplastic large cell lymphoma. Misclassification as anaplastic large cell lymphoma or another ALK-driven neoplasm could lead to inappropriate hematologic staging or systemic therapy; therefore, recognition of this immunophenotypic pattern has direct implications for patient management. To the best of our knowledge, ALK expression has been reported in only one previous case of IN-TSCT [3]. Interestingly, ALK immunoreactivity has also been documented in angiomatoid fibrous histiocytoma, another *EWSR1::ATF1* fusion-associated neoplasm, and in several other rare mesenchymal tumors harboring *EWSR1::ATF1* or related FET::CREB family fusions [4,5,6]. In angiomatoid fibrous histiocytoma, ALK expression may occur in the absence of *ALK* rearrangement or amplification, suggesting that ALK immunoreactivity is not necessarily a surrogate for an underlying *ALK* genomic alteration. As summarized in a recent review, EWSR1::ATF1 functions as an oncogenic transcription factor capable of inducing aberrant transcriptional programs [7]. Furthermore, fusion-driven transcriptional activation of *ALK* has been demonstrated in TFCP2-rearranged sarcomas [8]. Accordingly, because *EWSR1::ATF1* acts as an oncogenic transcription factor in other tumor contexts, aberrant transcriptional upregulation of *ALK* represents a possible explanation for the strong ALK immunoreactivity observed in the present case. However, this proposed mechanism remains hypothetical and should be regarded as an area for future investigation rather than an established biological explanation. Transcriptomic or functional validation will be required to clarify the basis of ALK expression in IN-TSCT. The patient remained free of local recurrence and distant metastasis 18 months after orchiectomy. Although this short-term outcome is favorable, IN-TSCT has been associated with aggressive behavior and metastatic spread in previous reports. Continued long-term oncologic surveillance is therefore warranted because the natural history and prognostic spectrum of IN-TSCT remain incompletely defined.

## Data Availability

The original contributions presented in this study are included in the article. Further inquiries can be directed to the corresponding author.

## Abbreviations

The following abbreviations are used in this manuscript:

AFP	Alpha-Fetoprotein
ALK	Anaplastic Lymphoma Kinase
β-hCG	Beta-Human Chorionic Gonadotropin
CD	Cluster of Differentiation
CT	Computed Tomography
EMA	Epithelial Membrane Antigen
FDG	Fluorodeoxyglucose
IN-TSCT	Inflammatory and Nested Testicular Sex Cord Tumor
LCA	Leukocyte Common Antigen
NGS	Next-Generation Sequencing
PET	Positron Emission Tomography
VAF	Variant Allele Frequency
